# Molecular Targets of Bis (7)-Cognitin and Its Relevance in Neurological Disorders: A Systematic Review

**DOI:** 10.3389/fnins.2019.00445

**Published:** 2019-05-09

**Authors:** Dalinda Isabel Sánchez-Vidaña, Jason Ka Wing Chow, Sheng Quan Hu, Benson Wui Man Lau, Yi-Fan Han

**Affiliations:** ^1^Department of Rehabilitation Sciences, The Hong Kong Polytechnic University, Hong Kong, China; ^2^Department of Applied Biology and Chemical Technology, The Hong Kong Polytechnic University, Hong Kong, China

**Keywords:** B7C, Alzheimer's disease, AChE inhibitor, cognitive impairment, NMDA antagonist

## Abstract

**Background:** The exact mechanisms involved in the pathogenesis of neurodegenerative conditions are not fully known. The design of drugs that act on multiple targets represents a promising approach that should be explored for more effective clinical options for neurodegenerative disorders. B7C is s synthetic drug that has been studied for over 20 years and represents a promising multi-target drug for the treatment of neurodegenerative disorders, such as AD.

**Aims:** The present systematic review, thus, aims at examining existing studies on the effect of B7C on different molecular targets and at discussing the relevance of B7C in neurological disorders.

**Methods:** A list of predefined search terms was used to retrieve relevant articles from the databases of Embase, Pubmed, Scopus, and Web of Science. The selection of articles was done by two independent authors, who were considering articles concerned primarily with the evaluation of the effect of B7C on neurological disorders. Only full-text articles written in English were included; whereas, systematic reviews, meta-analyses, book chapters, conference subtracts, and computational studies were excluded.

**Results:** A total of 2,266 articles were retrieved out of which 41 articles were included in the present systematic review. The effect of B7C on molecular targets, including AChE, BChE, BACE-1, NMDA receptor, GABA receptor, NOS, and Kv4.2 potassium channels was evaluated. Moreover, the studies that were included assessed the effect of B7C on biological processes, such as apoptosis, neuritogenesis, and amyloid beta aggregation. The animal studies examined in the review focused on the effect of B7C on cognition and memory.

**Conclusions:** The beneficial effects observed on different molecular targets and biological processes relevant to neurological conditions confirm that B7C is a promising multi-target drug with the potential to treat neurological disorders.

## Introduction

Neurodegenerative disorders are not only disabling but incurable conditions characterized by the loss of neurons leading to chronic degeneration and deterioration of the brain (Pérez-Hernández et al., 2016). Alzheimer's disease (AD), Parkinson's disease, Multiple sclerosis, Huntington's disease, and Amyotrophic lateral sclerosis are the major causes of death in the elderly population, and they have been projected to be the second leading cause of death by 2040 (Valera and Masliah, 2016). AD is the most common cause of dementia and the most frequent neurodegenerative disorder manifesting in the decline in cognition, memory ability, and language and problem-solving skills due to failure in the synaptic signal transfer, decreased number of synapses, and neuronal death (Baquero and Martín, 2015; Teipel et al., 2016).

The molecular mechanisms behind neurodegenerative diseases remain elusive (Santiago et al., 2017). However, it is clear that the multifactorial pathogenic nature of neurodegenerative disorders requires the use of several drugs to tackle the multiple symptoms present in these disorders (Li et al., 2009). Another therapeutic strategy to address the multifactorial complexity of neurodegenerative disorders is the search for compounds that interact with multiple targets known as multi-target design ligands (Van der Schyf, 2011; Ramsay et al., 2016). This strategy requires biological screening at the early stages of drug discovery and lead optimization to screen for drugs that can interact with two or more desired targets (Ramsay et al., 2016). The design of drugs that act on multiple targets represents a promising approach that should be explored for more effective clinical options for neurodegenerative disorders (Trippier et al., 2013). Drugs with multi-target properties have the potential to provide a more significant effect by acting on different brain regions relevant to the mechanism of disease and symptomatology of the disorder (Li et al., 2007b; Ramsay et al., 2016). The lack of effective treatment options that stop the progression of the disease and alleviate the multiple symptoms observed in this kind of disorders is driving the search for novel therapeutic alternatives.

Bis (7)-cognitin was first synthesized as part of a two-step prototype optimization strategy using computer modeling of ligand docking with target proteins. B7C showed to be highly potent, selective and cheap acetylcholinesterase inhibitor with promising therapeutic application in Alzheimer's disease (Pang et al., 1996). In order to evaluate the multi-target potential of B7C for the treatment of neurodegenerative disorders, a detailed analysis of the existing studies on B7C is inevitable.

Since its synthesis, many studies have been conducted to evaluate the mechanism of action of B7C using different cell-based platforms and animal models focusing the neurotherapeutic effect of B7C. In order to evaluate the potential clinical applications of B7C and assess its usefulness as a multi-target drug, it is crucial to carry out an in-depth analysis of the studies conducted of B7C. Therefore, the present systematic review aims at examining existing studies on the effect of B7C on different molecular targets and at discussing the relevance of B7C in neurological disorders. The research questions used as guidance for the study include: What molecular targets in the central nervous system have been identified for B7C? What is the relevance of B7C on neurological disorders based on the evidence from pre-clinical studies?

## Methods

### Search Strategy and Selection of Studies

Search terms were pre-defined, and a search strategy was established as shown in Table 1. The search terms were used to retrieve articles using the following databases: Embase, Pubmed, Scopus, and Web of Science. The settings for the database search included no restriction in terms of the year of publication, the advance search option was utilized for the combined search, and no filter was set in the database search. The retrieved articles from all the databases were pooled. The title of the articles was checked, and duplicate articles were removed. The titles of the remaining articles were screened to preselect relevant articles based on predefined inclusion and exclusion criteria described in the next section. Articles that did not fulfilled the inclusion criteria were removed. The full text of the preselected articles was read through for further article selection. Only articles that fulfilled the inclusion criteria were included in the systematic review. The database search and selection of the articles were carried out by two independent authors. Discrepancies in the selection were resolved by discussion, and a third author was involved as adjudicator where it was deemed necessary.

**Table 1:** Pre-defined search terms and database search strategy.

**ID**	**Search terms**
1.	Bis 7 cognitin
2.	B7C
3.	Bis heptyl cognitin
4.	Bis 7 tacrine
5.	B7T
6.	1 OR 2 OR 3 OR 4 OR 5

### Inclusion and Exclusion Criteria

Included are full-text studies written in English focusing on the effect of B7C on neurological disorders. Excluded are systematic reviews, meta-analyses, book chapters, conference abstracts, and computational studies.

### Data Extraction and Analysis

The data extracted from the selected studies included the type of study, the molecular target or behavioral test evaluated, and the conclusions. The cell line, *in vitro* assay, and concentration of B7C was extracted from the *in vitro* studies; whereas, the animal species, strain, and B7C dose used were extracted from the *in vivo* studies. The evidence of the effect of B7C on the respective molecular target was analyzed and the relevance on neurological disorders discussed.

## Results

**Figure 1:**
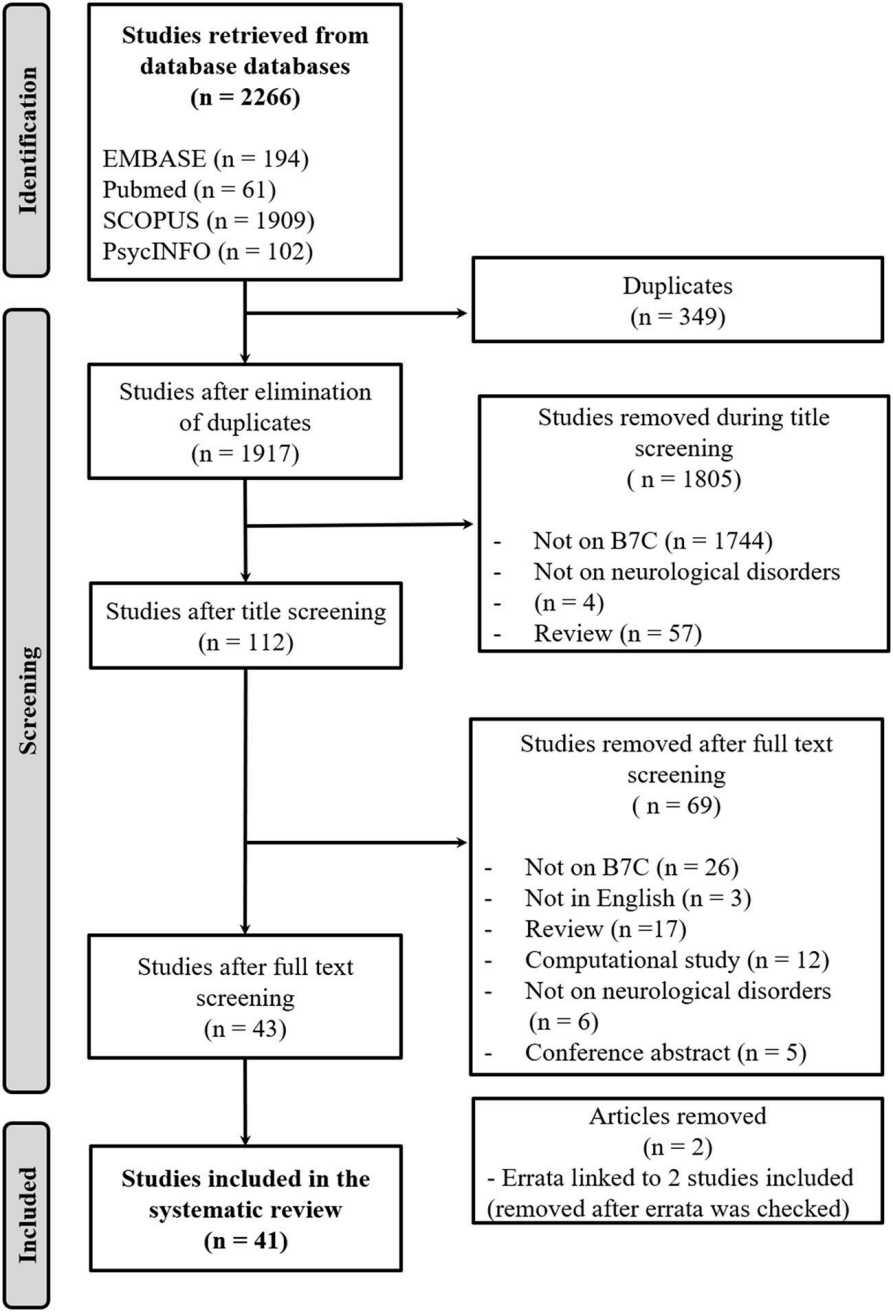
Study selection flow chart.

A total of 2266 articles were pooled from all the databases. The ratio was as follows, 194 articles from Embase, 61 from Pubmed, 1,909 from Scopus, and 102 from Web of Science. An overview of the flow chart with regard to the selection of articles is illustrated in Figure 1. A total of 349 articles were removed from the list due to their being duplicates. In the preselection stage, 1805 articles were omitted as they did not focus on B7C (*n* = 1744), were not concerned with neurological disorders (*n* = 4), or were review articles (*n* = 57). Out of the remaining 112 articles, 69 were excluded as they did not focus on B7C (*n* = 26), were not written in English (*n* = 3), did not focus on neurological disorders (*n* = 6), were review articles (*n* = 17), computational studies (*n* = 12), or conference abstracts (*n* = 5). Two of the preselected articles were an erratum linked to two studies that were included. The errata were checked and found to be referring to amendments in the author list, article title, or acknowledgments. Since the errata did not refer to relevant content in terms of the present systematic review, they were eliminated. A total of 41 articles fulfilled both the inclusion and exclusion criteria and thus made it into the final stage.

The data extracted from the selected studies are summarized in Table 2. Most of the studies that were included evaluated the effect of B7C on *in vitro* platforms (*n* = 25); whereas 13 studies evaluated the effect *in vivo*. *Three* studies included both *in vitro* and *in vivo* approaches. The molecular target that was primarily studied was AChE in 11 studies (Li et al., 1999; Wang et al., 1999b; Ros et al., 2001; Hu et al., 2002, 2015b; Fu et al., 2006; Yu et al., 2008; Pan et al., 2009; Bolognesi et al., 2010; Rizzo et al., 2011; Qian et al., 2014), followed by the NMDA receptor (*n* = 8) (Bai-fang et al., 2001; Li et al., 2005, 2007b; Luo et al., 2007; Liu et al., 2008a,b; Zhang et al., 2011; Liu and Li, 2012). The BChE was under scrutiny in five studies (Wang et al., 1999b; Hu et al., 2002; Bolognesi et al., 2010; Rizzo et al., 2011; Qian et al., 2014), the BACE-1 in four studies (Fu et al., 2008, 2009; Bolognesi et al., 2010; Rizzo et al., 2011), the GABA receptor in three studies (Li et al., 1999, 2007a; Zhou et al., 2009), the nitric oxide synthase (NOS) in two studies (Li et al., 2006, 2007b), the Kv4.2 potassium channels also in two studies (Nie et al., 2007; Li et al., 2010), the serotonin receptor 5-HT_3_ in one study (Luo et al., 2004), the α-secretase also in one study (Fu et al., 2009), the L-type voltage-dependent Ca^2+^ channels in one study (Fu et al., 2006), and the choline acetyl transferase in one study as well (Liu et al., 2000). Apoptosis (Han et al., 2000; Fu et al., 2007; Zhao et al., 2008; Fang et al., 2010), neuritogenesis, and neurite outgrowth (Chang et al., 2015; Hu et al., 2015a), long-term potentiation (Chang et al., 2015), amyloid beta (Aβ) aggregation and toxicity (Chang et al., 2015; Hu et al., 2015a), cell toxicity (Xiao et al., 2000), and retinal ischaemia (Li et al., 2014) were those biological processes where the effect of B7C was examined. The effect of B7C on spatial learning and memory was evaluated using the Morris water maze test in six studies (Wang et al., 1999a; Liu et al., 2000; Han et al., 2012; Shu et al., 2012; Chang et al., 2015; Hu et al., 2015b), the step-through task was used to measure the passive-avoidance response in two studies (Pan et al., 2007, 2009), the open field test was used also in two studies (Pan et al., 2009, 2011), and the novel object recognition test was used in one study (Han et al., 2012).

**Table 2:** Description of *in vitro* and *in vivo* studies on B7C.

**References**	***In vitro*** **study**		***In vivo*** **study**		**Molecular target, biological process or behavioral test**	**Conclusion**
	**Cell line**	**B7C concentration**	**Animal species/strain**	**B7C dose or concentration**		
Wang et al., 1999b	NA	NA	Female and male Sprague-Dawley rats	1, 3, 5, 9.5, 19, and 38 nM	AChE and BChE	-B7C inhibited both AChE (IC_50_ = 5.1 nM) and BChE (IC_50_ = 159 nM).
Ros et al., 2001	NA	NA	*Torpedo marmorata* (fish) electric organ Oocytes from mature females of *Xenopus laevis* (frog)	100 nM	ACh release by recording spontaneous synaptic activity	-B7C increased spontaneous quantal release from cholinergic terminals and blocked the AChE induced currents in a lower concentration than tacrine.
Hu et al., 2002	NA	NA	NA	Different concentrations	AChE and BChE	-B7C inhibited both AChE (IC_50_ = 2.7 nM) and BChE (IC_50_ = 2.6 nM).
Fu et al., 2006	Hippocampal pyramidal neurons (isolated from Wistar rats)	0.1, 1, 5, 10, 100, and 500 nM	NA	NA	AChE, L-type voltage-dependent Ca^2+^ channels	-B7C reduced the inward calcium current induced by Aβ induced Ca^2+^ which attenuated neuronal apoptosis.
Bolognesi et al., 2010	NA	NA	NA	Different concentrations	AChE and BChE (human recombinant enzymes) BACE-1, Aβ	-B7C inhibited both AChE (IC_50_ = 0.81 nM) and BChE (IC_50_ = 5.66 nM).
Rizzo et al., 2011	NA	NA	NA	Different concentrations	AChE and BChE (human recombinant enzymes), BACE-1	-B7C inhibited both AChE (IC_50_ = 0.81 nM) and BChE (IC_50_ = 5.66 nM) [results were taken as reference from Bolognesi et al. (2010)]. -B7C B7C inhibited BACE-1 (IC_50_ = 7.5μM), and inhibited AChE-induced Aβ aggregation [results were taken as reference from Fu et al. (2008)] (68%) [results were taken as reference from Bolognesi et al. (2010)].
Qian et al., 2014	Rat cortex homogenate/serum	0.5, 1, and 2 nM	NA	NA	AChE and BChE	-B7C inhibited both AChE (IC_50_ = 1.5 nM) and BChE (IC_50_ = 328.9 nM).
Hu et al., 2015b	SH-SY5S cells (human neuroblastoma cells)	0.1, 0.3, 1, 3, and 10 μM	Male Sprague-Dawley rats	0.1 and 0.2 mg/kg	AChE, ChAT, Morris water maze test	-B7C inhibited Aβ fibrils formation and disaggregated pre-formed Aβ fibrils. -B7C B7C reduced Aβ induced neurotoxicity. -B7C B7C inhibited the memory impairment induced by infusion of Aβ in rats. -B7C B7C reversed the dysfunction ChAT and AChE activity induced by Aβ in rats.
Pan et al., 2009	NA	NA	Male ICR mice	0.25–20 micromol/kg	AChE, OFT, and Step-Through task (passive-avoidance response)	-B7C inhibited AChE activity in brain tissue and serum 15 min after drug administration. -B7C B7C at 20 micromol/kg did not impair the open-field memory -B7C B7C reverted the scopolamine-induced learning and memory impairment at 1 micromol/kg. -B7C B7C was more potent than tacrine improving scopolamine-induced cognitive dysfunction.
Li et al., 1999	Hippocampal neurons (isolated from mice)	5, 25, 100 μM	NA	NA	AChE, GABA_A_	-B7C inhibited AChE (IC_50_ = 1.5 nM) -B7C B7C antagonizes the GABA_A_ receptor in a competitive mode.
Yu et al., 2008	NA	NA	Male ICR mice	3.54 micromol/kg	AChE	-B7C inhibited AChE (46.3%).
Fu et al., 2008	Mouse Neuro2a neuroblastoma cells	0.1, 0.3, 1, 2, and 3 μM	NA	NA	BACE-1	-B7C inhibits BACE-1, therefore decreases the generation of Aβ. -B7C B7C activates α-secretase.
Fu et al., 2009	Mouse Neuro2 neuroblastoma cells	0.1, 0.3, and 1 μM	NA	NA	BACE-1 and α-secretase.	-B7C inhibits BACE-1 and activates α-secretase.
Li et al., 2007a	DRG neurons (isolated from Sprague-Dawley rats)	Different concentrations	NA	NA	GABA receptor	-B7C binds to GABA receptor in a potent but reversible manner (IC_50_ = 6.28μM).
Zhou et al., 2009	Hippocampal neurons (primary cell culture from Sprague-Dawley rats)	1, 3, 5, 10, 30, and 100 μM	NA	NA	GABA_A_ receptor	-B7C is a competitive GABA_A_ receptor antagonist.
Luo et al., 2004	TG neurons		NA	NA	5-HT_3_ receptor	-B7C inhibited the 5-HT_3_ receptor current in a competitive manner.
Nie et al., 2007	DRG neurons (isolated from Sprague-Dawley rats)	10^−9^M to 10^−4^ M	NA	NA	Kv4.2 potassium channels	-B7C inhibited the delayed rectifier potassium channel and inhibited the Kv4.2 potassium channels. (IC_50_ = 0.72 μM).
Li et al., 2010	DRG (isolated from Sprague-Dawley rats)	1 μM	NA	NA	Kv4.2 potassium channels	-B7C suppressed the Kv4.2 potassium channels in a concentration-dependent manner (IC_50_ = 0.53 μM).
Bai-fang et al., 2001	Cortical cells (isolated from Sprague-Dawley rats)	0.3–1 μM/L	NA	NA	NMDA receptor	-B7C reduced the NMDA-mediated activity and exhibited a protective effect against glutamate-induced excitotoxicity.
Li et al., 2005	CGN (primary cell culture from Sprague-Dawley rats)	0.1 and 1 μM	NA	NA	NMDA receptor	-B7C prevents glutamate-induced neuronal apoptosis through blockade of the NMDA receptor -B7C B7C inhibits AChE and acts as NMDA antagonist. -B7C B7C inhibits the MAPK and ERK pathway.
Luo et al., 2007	Hippocampal neurons (isolated from Sprague-Dawley rats)		NA	NA	NMDA receptor	-B7C inhibited the NMDA receptor in a pH-dependent manner by desensitizing the receptors to proton inhibition.
Liu et al., 2008a	Hippocampal neurons (isolated from Sprague-Dawley rats)	0.001–1 μM	NA	NA	NMDA receptor	-B7C inhibited the NMDA receptor (IC50 = 0.68 μM). -B7C B7C prevented the glutamate-induced excitotoxicity by inhibition of the NMDA receptor.
Liu et al., 2008b	Hippocampal neurons (isolated from Sprague-Dawley rats)	0.5 μM	NA	NA	NMDA receptor	-B7C inhibited the NMDA receptors in a non-competitive manner and showed a protective effect against glutamate-induced neurotoxicity.
Zhang et al., 2011	RGC (primary cell culture from male Sprague-Dawley rats)	1 μM	Male Sprague-Dawley rats	0.05, 0.1, and 0.2 mg/kg	NMDA receptor	-B7C prevented NMDA-induced apoptosis in GCL. -B7C B7C inhibitory effect of NMDA receptors confers neuroprotection. -B7C B7C reduced the NMDA activated current in RGC which indicates that B7C is an antagonist of the NMDA receptor.
Liu and Li, 2012	HEK-293	1 μM	NA	NA	NR1, NR2A, and NR2B receptors	-B7C inhibited the NR1/NR2B receptor expressed in the cells in a slow onset, non-competitive and voltage-dependent manner which is similar to what is observed in rat hippocampal neurons expressing the NMDA receptors.
Li et al., 2007b	CGN (isolated from Sprague-Dawley rats)	Different concentrations	NA	NA	NMDA receptor, NOS	-B7C showed to be a moderated NMDA receptor antagonist and a selective NOS inhibitor.
Li et al., 2006	Cortical neurons (isolated from Sprague-Dawley rats)	0.001, 1 μM	NA	NA	NOS	-B7C reduced cell death induced by glutamate, Aβ, and L-arginine. -B7C B7C suppressed the activation of the NOS induced by glutamate. -B7C B7C inhibited the activity of NOS.
Liu et al., 2000	NA	NA	Male Sprague-Dawley rats	0.22, 0.44, and 0.89 micromol/kg/kg	ChAT, and the Morris water maze	-The induced learning and memory deficits were reversed by B7C in a dose-dependent manner.
Chang et al., 2015	Hippocampal neurons (isolated from Sprague-Dawley rats)	NA	Male ICR mice Wistar rats	0.1 or 0.2 mg/kg 0.1–3 μM	Morris water maze Neurite outgrowth LTP Aβ aggregation	-B7C reduced cognitive Aβ-induced cognitive impairment in mice at concentrations of 0.1 and 0.2 mg/kg -B7C B7C at a concentration of 0.1 to 0.3 μM prevented an Aβ-induced reduction in neurite length. -B7C B7C inhibited the formation of Aβ oligomers and reduced the amount of pre-formed oligomers. -B7C B7C showed a dose-dependent effect to prevent Aβ oligomer-induced inhibition of LTP with a threshold of 0.1 μM.
Han et al., 2000	Cortical astrocytes (isolated from ICR mice)	0.3, 1, 10, and 100 nM	NA	NA	Apoptosis	-B7C (1–10 nM) inhibited the ischemia-induced apoptosis.
Fu et al., 2007	CGN (isolated from Sprague-Dawley rats)	0.001, 0.01, 0.1, and 1 μM	NA	NA	Apoptosis	-B7C protected against the glutamate-induced excitotoxicity.
Zhao et al., 2008	NA	NA	Male Sprague-Dawley rats	0.05, 0.1, and 0.2 mg/kg	Apoptosis	-B7C showed anti apoptotic effect (0.2 mg/kg).
Fang et al., 2010	RGC (isolated from male Sprague-Dawley rats)	1 μM	Male Sprague-Dawley rats	0.05, 0.1, and 0.2 mg/kg	Apoptosis	-B7C inhibited glutamate-induced cell death (IC_50_ = 0.028 μM). -B7C B7C reduced glutamate-induced apoptosis *in vivo* (0.02 mg/kg).
Xiao et al., 2000	PC12 cells (rat pheochromocytoma cells)	0.01, 0.1, 1, and 10 μM	NA	NA	Cell toxicity	-B7C protected the cells against H_2_O_2_-induced cell toxicity improving the redox disequilibrium.
Hu et al., 2015a	PC12 cells (rat pheochromocytoma cells) Cortical neurons (isolated from Sprague-Dawley rats)	0.03–0.5 μM	NA	NA	Neuritogenesis Aβ neurotoxicity	-B7C induced neuritogenesis in PC12 and primary cortical neurons at concentrations of 0.1–0.5 μM and 0.1–0.3 μM, respectively. -B7C B7C (0.1–0.5 μM) provided neuroprotection against Aβ challenge in PC12 cells. -B7C B7C (0.1–0.5 μM) reverted the Aβ-induced neurite shortening in PC12 cells.
						-B7C acted via the activation of alpha7-nicotinic acetylcholine receptor/ERK pathway.
Li et al., 2014	NA	NA	Male Sprague–Dawley rats	0.2 mg/kg	Retinal ischemia	-B7C reduced ischemia-induced cell loss in the retinal ganglion cell layer showing a neuroprotective effect against ischaemic retinal damage.
Shu et al., 2012	NA	NA	Male Sprague-Dawley rats	0.2 mg/kg	Morris water maze test	-B7C decreased hippocampal neural apoptosis (it increased neurogenesis) in rats with chronic ischemia. -B7C B7C reversed the chronic-ischemia-induced decreased spatial learning and memory.
Wang et al., 1999a	NA	NA	Male Sprague-Dawley rats	0.18, 0.35, and 0.71 micromol/kg	Morris water maze test	-B7C ameliorated the memory deficits induced by scopolamine as observed in the decreased escape latency to levels compared to the ones in the control group in the Morris water maze test.
Han et al., 2012	NA	NA	Male Kunming strain mice	0.4, 0.5, and 0.6 micromol/kg	Morris water maze and the NOR task to evaluate recognition memory formation	-B7C mitigated the learning and memory deficits induced by scopolamine.
Pan et al., 2007	NA	NA	Male ICR mice	0.06, 1.25, 2.5, 5, 5, 10, and 20 micromol/kg	Passive avoidance response, spontaneous motor activity, hepatotoxicity	-B7C enhanced cognitive function at a high dose (20 micromol/kg) but produced motor dysfunction and hepatotoxicity.
Pan et al., 2011	NA	NA	Male ICR mice	0.25, 1, 5, and 20 micromol/kg	OFT	-B7C did not affect locomotion in the OFT. -B7C B7C (1 micromol/kg) improved the cycloheximide-induced amnesia in mice.

*Aβ, beta-amyloid; BACE-1, beta-secretase; BuChE, butyrylcholinesterase; ChAT, choline acetyltransferase; DRG, dorsal root ganglion; LTP, long-term potentiation; NA, not applicable; NOR, novel object recognition; NOS, nitric oxide synthase; OFT, open field test; RGC, retinal ganglion cells; TG, rat trigeminal ganglion; CGN, cerebellar granule neurons*.

The results of the studies that evaluated the effect of B7C on AChE and BChE demonstrated a clear inhibition of both enzymes with IC_50_ values ranging from 0.81 to 5.1 nM for AChE and from 2.6 to 328.9 nM for BChE, respectively. The antagonistic effect of B7C on the NMDA receptor was demonstrated in several studies. For example, treatment with B7C reduced the NMDA-mediated activity and showed a protective effect against glutamate-induced excitotoxicity (Bai-fang et al., 2001). Prevention of neuronal apoptosis by B7C blockade of the NMDA receptor was also observed (Li et al., 2005). Inhibition of the NMDA receptor by B7C was reported by Li et al. (2007b), Luo et al. (2007), Liu et al. (2008a,b), Zhang et al. (2011), and Liu and Li (2012). An inhibitory effect of B7C on BACE-1 led to a decrease in the generation of Aβ by activation of α-secretase (Fu et al., 2008, 2009; Bolognesi et al., 2010; Rizzo et al., 2011). Furthermore, B7C acted as a competitive antagonist of the GABA receptor (Li et al., 1999, 2007a; Zhou et al., 2009). Finally, treatment with B7C at doses of 0.18-0.89 μM/kg and 0.1–0.2 mg/kg showed improved cognitive performance in several animal models in which learning and memory deficits were induced, demonstrating the restorative effect of B7C (Wang et al., 1999a; Liu et al., 2000; Han et al., 2012; Shu et al., 2012; Chang et al., 2015; Hu et al., 2015b).

## Discussion

Tacrine (9-amino-1,2,3,4-tetrahydroacridine, under the trade name Cognex®, was the first drug approved for the treatment of AD in 1993 (Han et al., 2012). Other AChE inhibitors, such as donepezil (Aricept®), rivastigmine (Exelon®) and galantamine (Reminyl®) introduced in 1996, 2000, and 2001, respectively, in addition to the N-methyl-D-aspartate (NMDA) receptor antagonist memantine (Namenda®) followed the release of tacrine (Han et al., 2012; Lopes et al., 2017). Tacrine binds in a reversible mode to AChE and is considered a classical AChE pharmacophore (Lopes et al., 2017). Due to its side effects, such as hepatotoxicity and myopathy as well as its poor pharmacokinetic properties, including low bioavailability and narrow therapeutic index, a series of new tacrine-based compounds have been developed (Luo et al., 2004; Han et al., 2012). B7C, a product of the structure-activity-relationship drug design, is a dimer formed by two tacrine molecules linked by a spacer containing 7 methylene groups (Hu et al., 2015,a). This particular molecule has caught the attention of researchers, especially as a treatment option for AD. An advantageous feature of the B7C molecule is that it easily crosses the brain blood barrier due to its highly lipophilic profile making it a promising drug candidate for central nervous system disturbances (Hu et al., 2015a).

Although the pathological processes in AD are not well-understood, it is clear that disturbances in the cholinergic system and other neurotransmitters play a pivotal role in the pathogenesis of this neurodegenerative disorder (Han et al., 2012). Strategies to develop drugs for AD have focused on acetylcholinesterase (AChE) as a target for drug design based on the cholinergic hypothesis for AD (Ros et al., 2001; Lopes et al., 2017). The cholinergic hypothesis states that increased levels of acetylcholine in the brain alleviate the cognitive deficiencies observed in AD (Ros et al., 2001). Although a series of AChE inhibitors have been extensively studied, none of them represent a real cure for AD (Ros et al., 2001; Lopes et al., 2017).

The enzyme AChE has 2 sub-active sites within the binding pocket, namely the catalytic anionic site (CAS) and the peripheral anionic site (PAS) (Li et al., 2009). Using computational tools, molecular docking simulations were carried out to design and optimize the synthesis of tacrine analogs (Li et al., 2009). New compounds have been developed using the dual active sites in AChE as a basic hypothesis to increase the therapeutic action of tacrine analogs (Li et al., 2009). Alkylene-linked tacrine dimers interact with the CAS and PAS and potently inhibit AChE being B7C one of the promising alkylene dimers analogs (Lopes et al., 2017). As shown in Table 2, the multi-target activity of B7C has been evaluated in cell-based platforms and *in vivo*. The molecular targets studied included the AChE, BChE, NMDA receptor, ChAT, GABA receptor, BACE-1, K_v_4.2 potassium channels, NOS, and the 5-HT_3_ receptor. B7C has been found to be 150 times more potent and 250 times more selective to inhibit AChE when compared to tacrine (Ros et al., 2001; Li et al., 2009). The studies included in the present systematic review not only demonstrated the inhibitory effect of B7C on AChE, but also supported the promising action of B7C on neurodegenerative disorders based on the AChE hypothesis. The biological properties of B7C are superior to those of tacrine because B7C interacts simultaneously with the CAS and PAS of the enzyme (Li et al., 2009). The addition of the heptylene chain to the two tacrine molecules allows the dual interaction with the AChE binding sites, which explains its superior activity compared to tacrine (Bolognesi et al., 2010). B7C is a multi-target compound that shows promising biological activity, including inhibition of AChE, prevention of the aggregation of the β-amyloid (Aβ) protein, regulation of the downstream signaling mediated by the NMDA receptor, and inhibition of the nitric oxide synthase (NOS) signaling pathway (Zhang et al., 2011).

AChE is an important element of the cholinergic system that acts in the synaptic cleft by hydrolyzing the neurotransmitter acetylcholine (ACh) at central and peripheral levels (Colović et al., 2013). In addition to its function in cholinergic synapses, AChE plays a very crucial role of AChE relevant for the disease mechanism of AD because it accelerates the formation of Aβ formation in the Alzheimer's brain (Inestrosa et al., 1996). AChE is an important target in neurodegenerative disorders as its inhibition leads to accumulation of ACh by decreasing the ACh breakdown rate (Colović et al., 2013). Also, AChE stimulates Aβ fibrillogenesis through the formation of AChE-Aβ complexes, which is a characteristic feature in AD patients (Muñoz-Ruiz et al., 2005). Due to the nature of the AChE enzyme, dual binding is a highly desirable property for the design of AChE inhibitors, such as B7C (Li et al., 2009).

Several studies (see Table 2) have already demonstrated the effect of B7C on the inhibition of AChE in a selective manner and at lower concentration than tacrine (Li et al., 1999; Wang et al., 1999b; Ros et al., 2001; Hu et al., 2002, 2015b; Fu et al., 2006; Yu et al., 2008; Pan et al., 2009; Bolognesi et al., 2010; Rizzo et al., 2011; Qian et al., 2014). Drugs that inhibit AChE keep the ACh levels high in the synaptic cleft, thereby stimulating cholinergic transmission in regions of the forebrain that compensate for the loss of cells (Muñoz-Ruiz et al., 2005; Colović et al., 2013).

B7C has also been evaluated on the NMDA receptor and has been found to show an antagonistic effect (Mattson, 2000; Bai-fang et al., 2001; Li et al., 2005, 2007b; Luo et al., 2007; Liu et al., 2008b; Zhang et al., 2011; Liu and Li, 2012). Excitotoxicity significantly contributes to neuronal cell damage and death in neurodegenerative disorders (Lipton, 2004). Excitotoxicity is the result of overactivation of the NMDA glutamate receptor that leads to an excessive Ca^2+^ influx in the cell (Newcomer et al., 2000). Glutamate, the major excitatory neurotransmitter in the brain, is a crucial mediator involved in the normal functioning of the nervous system (Lipton, 2004). It is hypothesized that chronic exposure to elevated levels of glutamate or glutamate receptor hyperactivity triggers apoptotic pathways, a phenomenon of clinical relevance in disorders, such as Huntington's disease, Parkinson's disease, Multiple sclerosis, HIV-associated dementia, Amyotrophic lateral sclerosis, Glaucoma, and Alzheimer's disease (Lipton, 2004).

The link between Aβ formation, deposition, and AD pathogenesis has been well-established (Murphy and LeVine, 2010). The findings of the effect of B7C on AChE have also demonstrated inhibition of the Aβ fibrils formation and stimulated the disaggregation of pre-formed Aβ fibrils and improved memory impairment induced by Aβ (Fu et al., 2006; Bolognesi et al., 2010; Rizzo et al., 2011; Hu et al., 2015b). The protective effect of B7C against Aβ challenge reported in the studies included demonstrating the promising potential of B7C as treatment of AD. The BACE-1 is the enzyme responsible for the onset of the generation of Aβ. Therefore, it represents a very promising target for AD (Vassar et al., 2009). Aβ is the major component that occurs in neuritic plates found in AD (Chen et al., 2012). BACE-1 overexpression or hyperactivity is associated with the pathogenesis of AD while the opposite scenario has been found to have a neuroprotective effect (Chen et al., 2012). The findings of two studies, shown in Table 2, demonstrated an inhibitory effect of B7C on BACE-1 leading to a decreased generation of Aβ (Fu et al., 2008, 2009). Therefore, B7C showed a protective effect for the treatment of AD.

Cognitive impairment is a common symptom of neurological diseases, including AD (Bland, 2016), which warrants potential treatment options that act on improving represents a promising drug for the treatment of neurological disorders in which cognition is affected.

An important mediator of cell death is the N the cognitive impairment induced by a neuropathological condition as valuable alternatives. B7C improved cognition, special learning, and memory observed in several animal models (Pan et al., 2007, 2011; Han et al., 2012; Shu et al., 2012). Consequently, it OS, which has also been evaluated in B7C studies (Li et al., 2006, 2007b). In these studies, B7C was shown to have an inhibitory effect on NOS (Li et al., 2006, 2007a). Hyperactivity of NOS leads to excitotoxicity-mediated cell death. The enzyme is tethered to the NMDA receptor and gets activated by the influx of Ca^2+^ which increases the levels of NO associated with stroke and neurodegenerative diseases (Lipton, 2004). As B7C has demonstrated NMDA and NOS inhibitory activity, it represents a valuable candidate for the treatment of degenerative disorders.

Several studies on B7C focused on apoptosis as a target physiological mechanism (Han et al., 2000; Xiao et al., 2000; Fu et al., 2007; Zhao et al., 2008; Fang et al., 2010). Abnormal regulation of neuronal cell death has been associated with many neurological disorders (Mattson, 2000). Excessive death of one or more populations of neurons occurs as a result of disease or injury (Mattson, 2000). In AD, loss of hippocampal and cortical neurons is responsible for the symptomatology observed in this neurodegenerative disease (Mattson, 2000). The regulation of apoptosis involves several mediators, such as neurotrophic factors (inhibition) or glutamate (activation) (Mattson, 2000). Excessive glutamate-mediated activity increases the influx of Ca^2+^ leading to excitotoxicity and ultimately cell death (Mattson, 2000). As shown in Table 2, B7C provided protection against glutamate-induced excitotoxicity and free radical-induced damage (Han et al., 2000; Xiao et al., 2000; Fu et al., 2007; Zhao et al., 2008; Fang et al., 2010).

Another target for B7C is the GABA receptor (Li et al., 1999, 2007a; Zhou et al., 2009). GABA is the major inhibitory neurotransmitter in the central nervous system and functions primarily as a metabolite and a neurotransmitter (Zhou et al., 2009; Best et al., 2014). Together with the excitatory neurotransmitter glutamate, GABA is an important modulator of the inhibitory-excitatory balance that is essential for the proper functioning of the brain (Wu and Sun, 2015). Dysfunctions in the GABA system have been closely linked with neurological disorders, such as Huntington's chorea, epilepsy, AD, anxiety, and depression (Krogsgaard-Larsen, 1992; Kim and Yoon, 2017). The GABA_A_ receptor is a ligand-gated ion channel that regulates the influx of chloride ions, causing hyperpolarization in the postsynaptic neuron (Kim and Yoon, 2017) and mediating the fast inhibitory neurotransmission in the brain (Best et al., 2014). Changes in the concentration of endogenous modulator or in the composition of the GABA_A_ receptor lead to downregulation of the neuronal inhibition seen in pathological states (Nuss, 2015). B7C has shown to bind to the GABA receptor in a potent but reversable manner displaying a competitive antagonistic role (Li et al., 2007a; Zhou et al., 2009). Since innhibition of GABA has been linked to improvements in pathological states, drugs that regulate the GABA system, such as B7C are highly relevant in the treatment of neurological disorders.

## Limitations

Only one study out of the 41 studies included in the present systematic review reported adverse effect related to the treatment with B7C, namely hepatotoxicity. Due to the lack of information on side effects linked to B7C in the studies included, an overview of the safety of the drug could not be carried out. Thus, it is recommended to analyze the safety profile of B7C based on pre-clinical assessment in further studies.

## Conclusions

B7C is a computationally designed drug that has shown promising effects on several *in vitro* and *in vivo* platforms for AD and other neurodegenerative disorders. In the last two decades, numerous studies have focused on the evaluation of B7C on different targets. From the analysis presented, it is clear that B7C shows a superior activity when compared to its basic structure tacrine. Also, the beneficial effects observed on different molecular targets and biological processes relevant to neurological conditions confirm B7C's potency as a multi-target drug for the treatment of neurological disorders.

## Author Contributions

DIS-V was responsible for the database search, article selection, and manuscript writing. JKWC was responsible for the database search and article selection. BWML resolved discrepancies in the selection process, reviewed, and approved the manuscript, and SQH and Y-FH reviewed the manuscript.

### Conflict of Interest Statement

The authors declare that the research was conducted in the absence of any commercial or financial relationships that could be construed as a potential conflict of interest.
